# Lower Partial Plates

**Published:** 1903-09-15

**Authors:** John Baur


### LOWER PARTIAL PLATES.

BY JOHN BAUR, D.D.S.

A set of artificial teeth for the lower jaw, and especially a partial set, seems to be considered the *bete noir* of prosthetic dentistry by some practitioners. A well adjusted and well articulated lower denture may, however be made perfectly satisfactory to the patient.

Lower partial plates should preferably be constructed of gold, as this gives them a little more weight; they will, consequently, not be as easily displaced py the action of the tongue or cheek muscles. Strength is the main reason that calls for a metal denture. The majority of lower partial plates are made for the replacement of the lower molars, and either one or both bicuspids. To replace these with a vulcanite base, sufficiently strong to be of good service, would necessitate considerable bulkiness, where it passes around the back of the incisors and cuspids, and is therefore uncomfortable to the wearer.

Generally, all lower partial plates for repair are broken at this point. So, to make a strong and durable piece of work, the front part, at least, should be constructed of metal, but an entire metal plate is the best of all.

The first requisite, as in all other cases, is an accurate impression, always to be taken in plaster. In the degree that the impression is difficult to secure by the use of plaster, the more imperative is the indication for the employment of that material.

I usually take an impression (not being careful to get a good one), and run a model, then I take a tray that comes nearest fitting the case, and saw out that part where the remaining teeth are situated and fit it on the model. In this way I can exactly see where the tray needs trimming. Then build up the cut-out part with wax sufficiently high to be clear of the teeth when tray is placed over them, and leave sufficient room for the plaster to give strength to the impression. Mix the plaster somewhat thicker than for an upper impression, and let it get pretty hard before you attempt to remove it, so that where it breaks it will break with a sharp fracture, and you will be enabled to put each piece back in its place in the impression tray.

Where the ridge is not well defined, and not favorable to the retention of the piece without some form of attachment, gold clasps are made to grasp some of the teeth. If a metal bar is used they are soldered to the plate, and for vulcanite a spur is soldered to the clasp.

To construct the clasp, take an accurate plaster impression of the teeth to be clasped. A convenient way to do this is to bend a piece of cardboard into form and use it as an impression tray. It is filled with plaster and carried well down on the teeth, so as to secure an accurate impression of the entire tooth and cervical portion of the gums. When set, the impression is broken through in the middle which can easily be done with this pasteboard tray, removed and placed together and wired. Should there be an approximating tooth it can be filled up with mouldine. It is not necessary to dry the impression before pouring the melted Melott's metal, as the steam generated can readily escape through the sides of the impression. On this die the clasp is constructed out of clasp metal (24 or 26 gauge), depending on the length of the tooth to be clasped, a long tooth requiring a thinner gauge. After the clasps have been made on this die, they are fitted to the teeth, and if a metal base is used, it is trimmed until it goes into position without disturbing them, but still coming in contact with them. The clasp and plate being in position, take an impression in plaster and, when set, remove. If the clasp and plate do not come away with the impression, they should be removed and placed back in their respective positions, and invested carefully. When the impression is removed, borax and solder is placed in contact with the plate and clasp, and the two are soldered together. The clasps having been attached, the plate is again fitted in the mouth and a beeswax or modeling compound bite and impression taken. The case is then mounted on the occluding frame in the usual manner of any partial case. Teeth are now selected and ground to place; and right here is, where some make a mistake. The lower molars and also bicuspids should be so mounted that when a plumb line is dropped from the lingual border of the lingual cusps of the inferior molar, it will fall clear of the body of the jaw, and not through center of the edentulous ridge, as many artificial teeth are arranged.

If a good impression has been secured, and care is here taken and the teeth well articulated, you will have a well-fitting plate, one that will not be thrown out of line by lateral movements.

Some object to the use of clasps, the principal one being that they give lodgement for food, thereby inviting decay. This objection is not valid when the teeth and denture are gi-en the care and attention that ordinary hygienic conditions demand, while the comfort afforded the wearer, where these appliances are indicated, more than compensates for any danger that might arlse along the lines indicated.—*Texas Journal.*

